# Evaluation of coding-independent functions of the transcribed bovine aromatase pseudogene *CYP19P1*

**DOI:** 10.1186/1756-0500-7-378

**Published:** 2014-06-20

**Authors:** Marina Chwalisz, Rainer Fürbass

**Affiliations:** 1 Leibniz Institute for Farm Animal Biology (FBN), Wilhelm-Stahl-Allee 2, Dummerstorf 18196, Germany

**Keywords:** *CYP19A1*, Placenta, Granulosa, Gene-pseudogene interference

## Abstract

**Background:**

*CYP19A1* encodes the aromatase which catalyzes the final reaction of estrogen biosynthesis. The bovine genome also contains a non-coding copy of *CYP19A1*, the transcribed pseudogene *CYP19P1*. Whereas *CYP19A1* is transcribed in all estrogen-producing tissues, mainly in the placenta and gonads, the *CYP19P1* transcript so far was detected in the placenta. Strikingly, one sequence segment of both transcripts exhibits an exceptional high identity of 98%, which implies selective pressure and suggests some kind of function. Only recently, indeed, coding-independent functions of several transcribed pseudogenes were reported. Therefore, we analyzed *CYP19P1* and *CYP19A1* transcripts with the aim to detect clues for gene–pseudogene interference.

**Findings:**

The *CYP19P1* transcript was first examined *in silico* for the presence of microRNA coding sequences and microRNA targets. Further, to identify tissues where *CYP19P1* and *CYP19A1* transcripts are co-expressed, as a pre-requisite for transcript interference, expression profiling was performed in a variety of bovine tissues. Our *in silico* analyses did neither reveal potential microRNA coding sequences, nor microRNA targets. Co-expression of the *CYP19* loci was demonstrated in placental cotyledons and granulosa cells of dominant follicles. However, in granulosa cells of dominant follicles the concentration of *CYP19P1* mRNA was very low compared to *CYP19A1* mRNA.

**Conclusions:**

*CYP19P1* and *CYP19A1* transcripts might interfere in placental cotyledons. However, in granulosa cells of dominant follicles relevant interference between gene and pseudogene transcripts is unlikely to occur because of the very low *CYP19P1*/*CYP19A1* transcript ratio.

## Findings

### Background

*CYP19A1* encodes the aromatase which catalyzes the conversion of androgens to estrogens. During an earlier screening of a bovine placental cDNA library for *CYP19A1* clones, we also isolated cDNA clones of a homologous pseudogene, *CYP19P1*[1,2]. Gene and pseudogene transcripts exhibit major differences due to the loss of several exons in the *CYP19P1* transcript. Furthermore, numerous mutations in the pseudogene sequence produced multiple translational stop codons in all reading frames and thereby abrogated its protein-coding function. Strikingly, however, mutations are unevenly distributed. A sequence segment of 177 bp corresponding to exon 5 of *CYP19A1* is highly conserved, showing 98% identity, compared to 89% sequence identity in general [2]. Both *CYP19* loci are located on the same strand of chromosome 10, being separated by 20 kb of genomic DNA [3,4].

Pseudogenes for long were considered as defunct copies of functional genes. However, recent evidence suggests that some pseudogenes might exert coding-independent functions [5,6]. Interestingly, gene-pseudogene interference was demonstrated by selective knock-down of the *ABCC6P1* pseudogene, which led to a decreased transcription of its *ABCC6* parent gene [7]. This recent evidence prompted us to take up again our analysis of the *CYP19P1* pseudogene with the aim to detect clues of gene-pseudogene interference. To this end, transcripts were searched *in silico* for microRNA-coding sequences and microRNA targets. Further, expression profiles of *CYP19A1* and *CYP19P1* were analyzed in a variety of bovine tissues.

### Materials and methods

Samples from placentas, ovarian granulosa cells, fetal ovaries, endometria, adrenal glands and livers were collected from slaughtered cows in a local abattoir. Tissue samples were stored in RNAlater (Qiagen, Hilden, Germany) at -20°C. Granulosa cells were frozen in liquid nitrogen and stored at -80°C. Placental cotyledons and caruncles were separated manually. Despite careful separation, caruncle samples might contain traces of cotyledonary cells. Ovarian dominant and pre-ovulatory follicles collected before and after the LH-surge, respectively, were identified as described in [8]. Follicles were punctured with 18G needles and granulosa cells were aspirated. Total RNA was prepared using the NucleoSpin RNA II Kit (Macherey-Nagel, Düren, Germany), according to the supplier´s protocol. This procedure included on-column DNaseI digestion to remove traces of DNA. RNA was quantified in a NanoDrop 1000 spectrophotometer (PeQLab, Erlangen, Germany). RNA integrity was confirmed by denaturing agarose gel electrophoresis.

**Figure 1:**
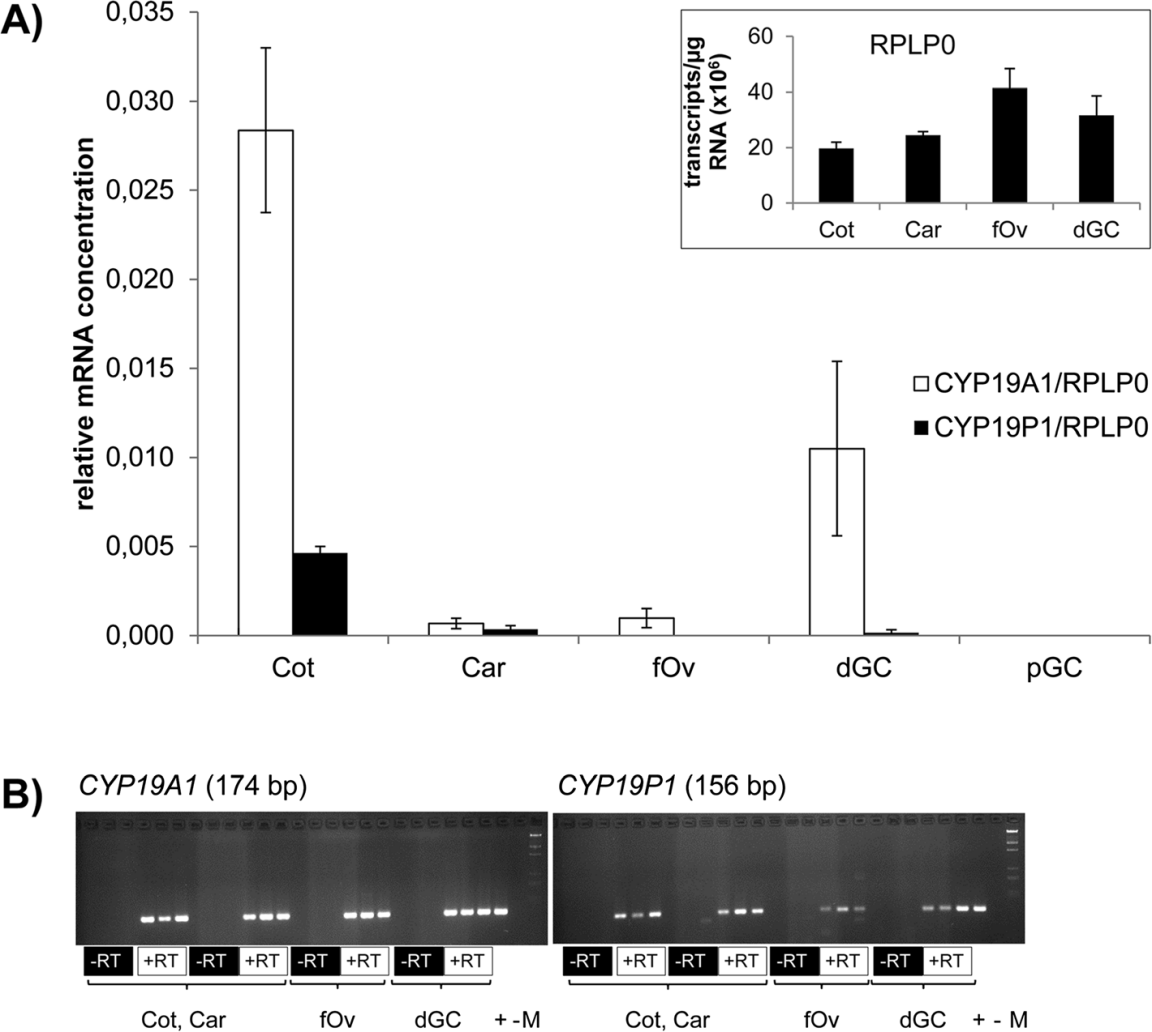
**Expression of *CYP19P1 *and *CYP19A1 *in bovine tissues. A)** Quantitative reverse transcription PCR analysis of *CYP19P1* and *CYP19A1* transcripts. Concentration values were normalized using *RPLP0* as an internal control. The abundance of the *RPLP0* transcript in the bovine tissues is indicated in the inserted diagram. The results are shown as means +/- SEM of n = 3 independent experiments. Analyzed tissues were placental cotyledons (Cot) and caruncles (Car), fetal ovaries (fOv) and granulosa cells from dominant and pre-ovulatory follicles (dGC and pGC, respectively). *CYP19A1* and *CYP19P1* transcripts were not detected in pGC, adrenal glands, endometria and livers. **B)** Agarose gels confirming the generation of only the expected products during the qPCR analysis shown in the panel A. The products of qPCR reactions including reverse transcription are indicated below the gels by white boxes labeled + RT. Control PCR reactions without prior reverse transcription (indicated by black boxes labeled –RT) did not yield products. Lanes labeled with **+**, **-** and **M** contain positive PCR controls (PCR products from cloned cDNAs), negative PCR controls (PCR reactions without templates) and molecular weight markers, respectively.

The abundance of *CYP19P1* and *CYP19A1* transcripts was measured by quantitative reverse transcription PCR (qPCR). For normalization purposes, the *RPLP0* transcript encoding a ribosomal large subunit protein was also measured. RNA (100 ng) was reverse transcribed in a 25 μl reaction volume using a mixture of random hexameric and oligo dT primers (Roche, Mannheim, Germany) and M-MLV reverse transcriptase (Promega, Mannheim, Germany). cDNA was purified with the High Pure PCR Product Purification Kit (Roche). For subsequent real time PCR, cDNA was amplified in a 12 μl reaction volume with the SensiFast SYBR No-ROX Kit (Bioline, Luckenwalde, Germany) using following primer pairs: ***CYP19P1*** **_for**, 5´-TCATTACAACGCATCCCCAGGTTGA-3´/***CYP19P1*** **_rev**, 5´-CTAGGTCCATGACGGGCTGGTATCA-3´, ***CYP19A1*** **_for**, 5´-GGATCGGCAGTGCCTGCAATTACTA-3´/***CYP19A1*** **_rev**, 5´-ATGCCGATGAACTGCAACCCAAGTT-3´ and **RPLP0_for**, 5´-TGGTTACCCAACCGTCGCATCTGTA-3´/**RPLP0_rev**, 5´-CACAAAGGCAGATGGATCAGCCAAG-3´ (Sigma-Aldrich, Taufkirchen, Germany). The primer pairs were designed to flank intronic sequences of genomic DNA. The expected products from cDNA were 156, 174 and 140 bp in length, respectively. The amplification and quantification of resulting PCR products were performed in a Light-Cycler 480 instrument (Roche) under following cycling conditions: Pre-incubation at 95°C for 5 min, followed by 40 cycles of denaturation at 95°C for 20 s, annealing at 60°C for 15 s, and extension at 72°C for 15 s, and single point fluorescence acquisition at 75°C for 10 s to avoid quantifying primer artifacts. The generation of only the expected products was confirmed by melting curve analysis and agarose gel electrophoresis, which did not reveal PCR products from genomic DNA (Figure 1B). External standard curves were generated by co-amplification of various dilutions of cloned PCR products (5 × 10^−12^ to 5 × 10^−16^ g DNA/reaction) with the corresponding primer pairs.

The statistical analyses were performed with the Sigma Plot 12.0 Analysis System (Jandel Scientific, San Raffael, CA, USA).

### Results and discussion

The remarkably high conservation of *CYP19P1* in the exon 5-homologous sequence segment (henceforth referred to as P1-exon 5) implies selective pressure. Hence, it might well be that the pseudogene exerts a coding-independent biological activity via the P1-exon 5. Because pseudogene-derived small interfering RNAs were shown to regulate gene expression in mouse oocytes [9], we examined if also the *CYP19P1* transcript encodes microRNAs. We performed *in silico* analyses of the P1-exon 5 using the free software RNAfold [10] to detect putative stable pre-microRNA hairpins. However, no such structures were predicted by the program. The cellular abundance of the tumor suppressor gene *PTEN* transcript was found to be regulated by the transcript of the highly homologous *PTENP1* pseudogene via competition for microRNA binding [11]. To evaluate if the *CYP19* gene pair could also interfere this way, we searched the P1-exon 5 for microRNA target sites using the MIRANDA software [12]. However, probable targets of known microRNAs were not found. Other pseudogenes are transcribed in an antisense orientation and lead to silencing of their parent genes by translational interference [5]. However, this mode of action can not apply to *CYP19P1* and *CYP19A1* which are both encoded by the same strand of chromosome 10 [4] and hence are transcribed in the same orientation.

**Figure 2:**
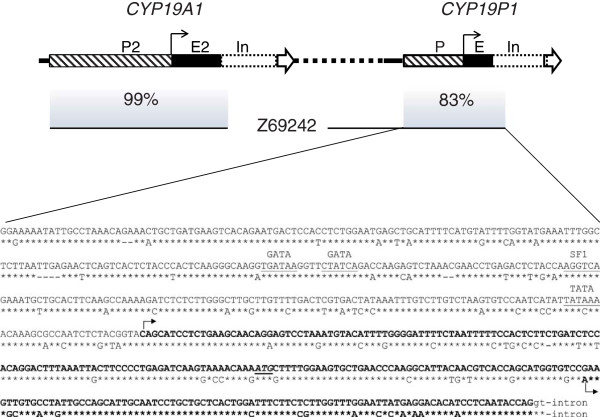
**Analysis of the putative *CYP19P1 *promoter.** The upper panel depicts a part of the bovine chromosome 10 (included in the GenBank sequence NW_003104282) with *CYP19A1* and *CYP19P1* depicted as arrow-headed boxes. The stippled line signifies 20 kb of intergenic DNA. Promoters (P2, P), exons (E2, E) and introns (In) are highlighted by hatching, black and white staining, respectively. Transcription start sites are represented by black arrows. The *CYP19A1* GenBank sequence Z69242 (black horizontal lines below the chromosome 10 schematic) covers the proximal promoter P2, exon 2 with the translation start codon, and part of intron 2. By BLAST analysis, two homologous regions were found in the NW_003104282 sequence (shaded areas). The lower panel shows aligned sequences of homologous *CYP19A1* and *CYP19P1* regions. Asterisks indicate identical bases, dashes are arbitrarily inserted for optimal alignment. Transcribed sequences are printed in bold and transcription start sites are marked by arrows. The translation start codon is underlined and printed in italics. Above the *CYP19A1* promoter sequence binding motifs of transcription factors GATA, SF1 and a TATA box are shown.

For possible interference, *CYP19P1* and *CYP19A1* transcripts need to be co-expressed. *S*o far, expression of *CYP19P1* has not been studied in bovine tissues other than placenta. To evaluate co-expression as a prerequisite for transcript interference, we performed qPCR to measure the abundance of *CYP19P1* and *CYP19A1* transcripts in a variety of bovine tissues. These included granulosa cells from dominant follicles and placental cotyledons, which were known to express *CYP19A1*[13]. Further, granulosa cells from pre-ovulatory follicles, fetal ovaries, placental caruncles, endometrium, adrenal gland and liver were also analyzed, to find out whether the tissue-specificity of *CYP19A1* expression is retained in *CYP19P1*. The results of the qPCR experiments are presented in Figure 1A. As expected, cotyledons and granulosa cells from dominant follicles expressed considerable amounts of the *CYP19A1* transcript. In contrast, the *CYP19A1* transcript concentration was low or undetectable in the remaining tissue samples. Likewise, the *CYP19P1* transcript was found in cotyledons and granulosa cells from dominant follicles, however, at a very low concentration. The ratio *CYP19P1*/*CYP19A1* of mean transcript concentrations was 0.16 and 0.016, respectively. Considering these results *CYP19P1* and *CYP19A1* transcripts might interfere in placental cotyledons, although the underlying mechanism remains unclear. On the other hand it is unlikely that the *CYP19P1* transcript plays a major role in the regulation of *CYP19A1* expression in granulosa cells of dominant follicles. Interestingly, however, in granulosa cells from pre-ovulatory follicles *CYP19P1* expression is shut down, just as known from *CYP19A1*, as a consequence of the pre-ovulatory LH-surge [14,8]. The apparent congruent tissue-specificity of *CYP19* gene and pseudogene could be explained by promoter conservation. A genomic DNA sequence covering the entire *CYP19A1* and *CYP19P1* loci is present in the database [GenBank:NW_003104282]. We defined the genomic sequence immediately upstream of the transcribed *CYP19P1* sequence as potential pseudogene promoter and compared it with promoters of *CYP19A1* by BLAST analysis. Thereby we found, that 483 bp of the proximal *CYP19A1* promoter 2 ( [15]; [GenBank:Z69242]) were duplicated during pseudogene genesis (Figure 2). We have verified the sequence by sequencing cloned PCR products (data not shown). Gene and pseudogene promoters exhibit 83% sequence identity. Although binding motifs of GATA and SF1 transcription factors are retained, the putative pseudogene promoter has lost the functional TATA box, which might explain the low promoter activity and the use of an alternative transcription start site.

## Competing interests

The authors declare that they have no competing interests.

## Authors’ contributions

MC implementation, analysis of data and preparation of the manuscript. RF initiation of the study, analysis of data, preparation of the manuscript. Both authors read and approved the final manuscript.
